# Understanding the Role of Different Substrate Geometries for Achieving Optimum Tip-Enhanced Raman Scattering Sensitivity

**DOI:** 10.3390/nano11020376

**Published:** 2021-02-02

**Authors:** Lu He, Mahfujur Rahaman, Teresa I. Madeira, Dietrich R.T. Zahn

**Affiliations:** Semiconductor Physics, Chemnitz University of Technology, D-09107 Chemnitz, Germany; lu.he@s2017.tu-chemnitz.de (L.H.); Teresa.madeira@physik.tu-chemnitz.de (T.I.M.); zahn@physik.tu-chemnitz.de (D.R.T.Z.)

**Keywords:** gap-mode TERS, FEM simulations, metallic nanostructures, plasmonic modes, enhancement factor, spatial resolution

## Abstract

Tip-enhanced Raman spectroscopy (TERS) has experienced tremendous progress over the last two decades. Despite detecting single molecules and achieving sub-nanometer spatial resolution, attaining high TERS sensitivity is still a challenging task due to low reproducibility of tip fabrication, especially regarding very sharp tip apices. Here, we present an approach for achieving strong TERS sensitivity via a systematic study of the near-field enhancement properties in the so-called gap-mode TERS configurations using the combination of finite element method (FEM) simulations and TERS experiments. In the simulation study, a gold tip apex is fixed at 80 nm of diameter, and the substrate consists of 20 nm high gold nanodiscs with diameter varying from 5 nm to 120 nm placed on a flat extended gold substrate. The local electric field distributions are computed in the spectral range from 500 nm to 800 nm with the tip placed both at the center and the edge of the gold nanostructure. The model is then compared with the typical gap-mode TERS configuration, in which a tip of varying diameter from 2 nm to 160 nm is placed in the proximity of a gold thin film. Our simulations show that the tip-nanodisc combined system provides much improved TERS sensitivity compared to the conventional gap-mode TERS configuration. We find that for the same tip diameter, the spatial resolution achieved in the tip-nanodisc model is much better than that observed in the conventional gap-mode TERS, which requires a very sharp metal tip to achieve the same spatial resolution on an extended metal substrate. Finally, TERS experiments are conducted on gold nanodisc arrays using home-built gold tips to validate our simulation results. Our simulations provide a guide for designing and realization of both high-spatial resolution and strong TERS intensity in future TERS experiments.

## 1. Introduction

Tip-enhanced Raman spectroscopy (TERS) is a powerful technique that combines Raman spectroscopy with scanning probe microscopy (SPM), i.e., atomic force microscopy (AFM) or scanning tunneling microscopy (STM). TERS thus probes the local vibrational properties of analytes with a spatial resolution far beyond the diffraction limit of incident light. When excited with a suitable light source, the sharp metallic tip confines and enhances the optical field in the vicinity of the tip apex, which then produces Raman scattering from a nanoscopic volume of a sample under the tip. Therefore, by scanning the tip across the sample surface one can realize Raman images of the materials to be probed with an excellent spatial resolution. For example, a resolution of 1.7 nm for carbon nanotubes [1], 3 nm for a Pd/Au bimetallic model catalyst [2], and 2.3 nm for MoS_2_ [3] in ambient conditions using AFM-TERS and sub-nanometer resolution [4,5,6] for probing benzene rings via STM-TERS in ultra-high vacuum were reported.

TERS works based on the principle of electromagnetic (EM) field enhancement around the tip apex, since a metal nanostructure would employ the localized surface plasmon resonance (LSPR) and the lightning rod effect [7]. The lightning rod effect is introduced by the geometric anisotropy of the tip and is independent of the excitation wavelength, while the LSPR is created due to the collective oscillation of free charge carriers in the metal nanostructure [8]. There are many factors that influence the LSPR, such as material, size, shape, and morphology [9]. Therefore, an appropriate selection/modification of these parameters can tune the LSPR over a wide spectral range.

Gold and silver are the two most widely used noble metals for TERS tips due to their small dielectric loss [10], stability in air, and tunability of the LSPR in the visible spectrum. Both metals have some advantages and disadvantages, which become the critical deciding factors for TERS applications. While silver shows a more pronounced plasmonic effect, gold provides a better environmental stability. Consequently, the latter makes gold the more popular material for TERS tips. The two main parameters of TERS compared to conventional Raman spectroscopy are the enhanced signal intensity (also known as enhancement factor (EF)) and the spatial resolution (both together we termed as TERS sensitivity in this work). A usual practice of increasing TERS sensitivity multi-fold compared to conventional TERS is to introduce a metal substrate in order to obtain the so-called gap-mode TERS configuration [11,12]. In this configuration, an image dipole is formed in the metal substrate, thus increasing the sensitivity to a great extent. The EF in a gap-mode TERS experiment can reach up to 10^6^–10^8^ [13] and can be three orders of magnitude higher than that of non-gap-mode TERS [14]. The width of the confined field in the gap-mode TERS configuration is strongly related with the tip radius and the tip-sample gap [9,15,16]. Interestingly, as reported by Xu et al. [9], there is a compromise between the enhancement factor (EF) and the spatial resolution with respect to the tip diameter at resonant excitation. A larger tip radius provides better signal intensity due to the larger scattering cross section at the cost of decreased spatial resolution. Therefore, in order to achieve a better spatial resolution, one needs to decrease either the tip radius or the tip-sample gap. Since decreasing the tip-sample gap below 1 nm induces non-local quantum effects, which quenches the TERS sensitivity [17], decreasing the tip radius is a more straightforward method to obtain higher TERS sensitivity via combination of LSPR and the lightning rod effect with very little trade-off [18].

However, fabricating sharp tips with good reproducibility to achieve a considerable lightning rod effect is still a big challenge [19,20,21,22,23,24,25,26]. On the other hand, high spatial resolution together with marked EF can be obtained using a metal nanostructured substrate as demonstrated in our previous work [3]. This is advantageous since the fabrication techniques of plasmonic substrates in a reproducible manner with controllable sizes and shapes are well established [27,28]. Using such an approach, we can tune the combined tip-substrate plasmonic system with a wide range of parameters and spectral range customized to an existing experimental configuration. Even though some recent works addressed the influence of the substrate on the TERS sensitivity [29,30], a systematic understanding of the effect of substrate size and shape on the TERS sensitivity is still missing. In this work, we at first perform a series of 2D numerical investigations on the evolution of the local enhancement factor and the spatial resolution with respect to the typical gap-mode TERS geometry that consists of a gold tip with varying apex diameter from 2 nm to 160 nm on flat gold. Then, a gold nanodisc of 20 nm height on a flat gold substrate with the nanodisc diameter varying from 5 nm up to 120 nm serves as a model for a nanostructured substrate. In this case, the gold tip diameter is fixed at 80 nm. Finally, TERS experiments are performed and the results are compared with the corresponding 3D simulation.

## 2. Materials and Methods

### 2.1. 2D Numerical Methods and Geometries

**Figure 1:**
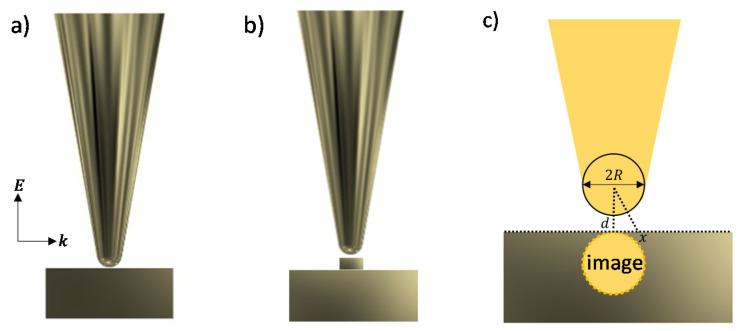
Simulated tip-enhanced Raman spectroscopy (TERS) models consists of (**a**) conical gold tip with various diameters from 2 nm to 160 nm placed on a flat gold film substrate and (**b**) conical gold tip with fixed diameter of 80 nm and a flat gold substrate modified by a 20 nm high gold nanodisc with varying diameter from 5 nm to 120 nm. (**c**) Schematic representation of the image dipole formation from the model shown in Figure 1a. The wavevector propagates along the *x*-axis and the $\vec{E}$ vector is along the *y*-axis.

All tip-substrate systems considered in this study are simulated with a tip-substrate gap of 1 nm, below which quantum effects may dominate [31]. Finite element method (FEM) simulations are performed in a 2D model to avoid the huge computational cost by 3D simulations in the frequency domain of Wave Optics Module using the COMSOL Multiphysics® software v.5.6. Nevertheless, a few representative 3D models are also simulated for comparison with specific 2D simulation and experimental results. The electric field is calculated by solving Maxwell’s $\nabla \times \mu_{r}^{-1}\left(\nabla \times \vec{E}\right)-k_{0}^{2}\left(\varepsilon_{r}-\frac{j\sigma }{{\omega \varepsilon }_{0}}\right)\vec{E}=0$, with $\vec{E}\left(x,y,z\right)=\tilde{E}\left(x,y\right)e^{-ik_{z}z}$. The incident optical field is set as a plane wave propagating along the *x*-axis, with $\vec{E}$ varying along the *y*-axis as indicated in Figure 1.

The geometry shown in Figure 1a consists of a conical tip with varying tip apex (diameter from 2 nm to 160 nm) and a flat gold thin film substrate. This geometry is representative for conventional gap-mode TERS and is thus simulated to benchmark our simulations as this configuration was already studied in detail [9,32,33,34]. To investigate the influence of the substrate geometry on TERS sensitivity, a gold nanodisc having a 2D-cross section of rectangular shape is added to the flat gold thin film with its diameter varying from 5 nm to 120 nm (see Figure 1b). The height of such nanodisc is fixed at 20 nm to be consistent with the following TERS experiment. Moreover, the edge of such a nanostructure in a real situation should always possess some curvature rather than a single sharp point. Therefore, we set a roundish shape at the corner of the rectangle with a constant curvature of 2 nm. The diameter of the conical metal tip apex is fixed at 80 nm in this configuration to be consistent with the experiment.

A rectangular perfect matched layer (PML) is used in all simulations to absorb the light scattered from the boundary [35]. The amplitude of the electric field of the excitation light is set at 1 V/m. An optimized mesh is used after a convergence test in COMSOL, and the minimum element size in the gap area is set at 0.1 nm. A non-uniform mesh is used in the remaining calculated area to balance the performance and the computation time with the maximum element size set as λ/5n, in which λ is the excitation wavelength and n is the refractive index. The refractive index of gold used in the simulations is taken from ref. [36].

The spectral dependence is obtained via a wavelength sweep from 500 nm to 800 nm for each specific configuration. The maximum TERS EF ($E_{loc}^{4}/E_{0}^{4})$ for each configuration is taken under the resonance condition where the local field has the maximum spectra-dependent value. The local electric field enhancement factor is calculated at the middle point of the tip-substrate gap.

An analysis of the spatial resolution could be performed using the analogue sketch shown in Figure 1c, which represents the conventional gap-mode TERS configuration as shown in Figure 1a. The drop in potential between the two spheres (original and image dipoles) can be expressed as $∆V=\left|E_{loc}\right|d$, while the potential difference between these two sites in the absence of two metal spheres can be expressed as $∆V=\left|E_{0}\right|\left(2R+d\right)$, where $E_{0}$ is the incident field and $E_{loc}$ is the local field. Since the two spheres can be considered as equipotential bodies, we can write $∆V=\left|E_{0}\right|\left(2R+d\right)=\left|E_{loc}\right|d$. In a certain specific geometry where R and d are fixed. Thus, the lateral offset of electric field from the center can be written as

(1) $$\left|E_{loc}\left(x\right)\right|=\frac{∆V}{2R+d-2\sqrt{R^{2}-x^{2}}}$$

The full-width-at-half-maximum (FWHM) of the local field is thus given by $w=2\sqrt{Rd}$ [15]. 

Using a similar approach considering the TERS intensity as the 4th power of the local field amplitude, we can write the lateral offset of the TERS signal as

(2) $${\left|E_{loc}\left(x\right)\right|}^{4}=∆V^{4}/{\left(2R+d-2\sqrt{\left(R^{2}-x^{2}\right)}\right)}^{4}$$

Therefore, for a very small d, we can obtain the FWHM of the TERS intensity distribution as

(3) $$\mathrm{FWHM}=2\sqrt{\left(\left(\sqrt[{4}]{2}-1\right)Rd\right)}\approx 0.87\sqrt{Rd}$$

### 2.2. 3D Numerical Methods and Models

3D simulations employing the TERS experimental geometry are performed for comparison with the 2D simulation and the experimental results. The geometry consists of a conical gold tip with a diameter of 80 nm either placed on a flat gold substrate or on the edge of a gold nanodisc with height of 20 nm and diameter of 100 nm. 638 nm excitation is used at an incident angle of 65° in the xz-plane. The light is a *p*-polarized plane wave with its polarization direction and the wavevector are set to be parallel and perpendicular to the tip along axis, respectively. The optical constants of gold used are the same as in the 2D model. An optimal mesh is used with the minimum element size of 0.5 nm. A perfect matched layer is used to absorb the light scattered from the boundary.

### 2.3. Experimental Materials and Methods

TERS experiments are performed using home-made gold coated tips. For this purpose, 100 nm of gold is evaporated onto commercially available silicon tips using thermal evaporation resulting in the formation of gold clusters at the tip apex. Details of the tip preparation can be found in ref. [37]. The typical diameter of these clusters at the apex are between 80 and 120 nm. The substrate consists of gold nanodiscs on a continuous gold film of 50 nm on a Si substrate. The diameter of the nanodiscs is 100 nm with a height of 20 nm and the distance between two nanodiscs from centre to centre is 150 nm. An ultrathin film of 2 nm cobalt phthalocyanine (CoPc) is deposited on the plasmonic substrate as Raman probe using organic molecular beam deposition. 

TERS mapping is performed on a NanoRaman Platform from HORIBA Scientific (formerly AIST-NT) in side-illumination geometry using a wavelength of 638 nm for excitation. Incident and scattered light are collected by a long working distance objective (100×, 0.7 N.A.) inclined at an angle of 65° with respect to the normal of the sample surface. During the TERS measurements the AFM tip operates in intermittent contact mode while the TERS signal is acquired when the tip is in contact with the sample and the contact time is defined by the acquisition time of 0.3 s. An electron-multiplying charge-coupled device (CCD) is used to collect the scattered light signal dispersed by a 600 L/mm grating. The step size and the laser power used to acquire a TERS map is 10 nm and 1 mW, respectively.

## 3. Results and Discussion

### 3.1. 2D Simulation Results

#### 3.1.1. Simulation Results on Conventional Gap-Mode TERS Configuration Shown in Figure 1a

Starting with the conventional gap-mode TERS configuration (see Figure 1a), a gold tip with varying diameter from 2 to 160 nm is placed on a flat gold substrate. This gap-mode TERS configuration is a well-studied system using FEM simulations and a handful publications can be found investigating the effect of different parameters on TERS enhancement [9,32,33,34]. Surprisingly, we did not find any systematic study of the influence of the tip diameter on the spatial resolution.

**Figure 2:**
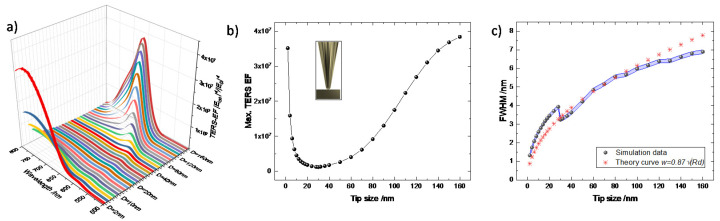
Simulation results of (**a**) the spectral dependence of the TERS EF, (**b**) max. TERS EF, and (**c**) full-width-at-half-maximum (FWHM) of the local TERS profile in the conventional gap-mode TERS geometry as shown in the inset of Figure 2b. The simulated FWHM as a function of the tip diameter is compared with the FWHM profile derived from Equation (3) (red asterisks in Figure 2c). The blue shaded area presents the error bar of the FWHM.

Figure 2 shows our simulation results for the conventional gap-mode TERS configuration. The overall spectral dependence of the TERS enhancement with respect to the tip diameter is presented in Figure 2a. The corresponding maximum TERS EF, i.e., TERS EF value at the resonance wavelength, becomes high for both very small and very large tip diameters, as can be clearly seen in Figure 2b. As the tip diameter increases from 30 nm to 160 nm, the TERS enhancement increases strongly. This trend of increasing TERS EF is due to the combination of two counter-effects, namely increasing scattering cross section and radiative damping, especially at higher tip diameters [38,39,40]. The gradual red shift of the max. TERS EF position also takes place in this range and originates from the size dependent LSPR effect similar to that observed in previous works [41,42,43]. In the lower tip diameter regime, the TERS EF increases at a much faster rate with decreasing tip diameter from 28 nm to 2 nm. The sharp rise of TERS EF is due to the lightning rod effect, because of a geometrical singularity-induced spatial confinement of the surface charge density at the apex. Our 2D simulation results are qualitatively in agreement with previous reports simulating similar geometries using a 3D-FDTD method [9]. In their study, the tip radius-dependent maximum electric field enhancement initially experiences a rapid decrease as the tip diameter increases from 10 nm to 30 nm (in our simulation from 2 nm to 30 nm) due to the lightning rod effect. Afterwards, the local electric field increases slowly from 30 nm to 100 nm (in our simulation from 30 nm to 160 nm). Further increase of the tip diameter does not provide additional enhancement, as it saturates beyond 100 nm in their study. However, we do not reach saturation in our 2D simulations. This could be due to the different geometry used in our 2D model and the 3D model simulated in the ref [9].

Interestingly, the resonance wavelength in the lightning rod effect regime shows a red shift with decreasing tip diameter in agreement with previous work [44,45,46,47]. This might be due to the electrical conductivity of the tip material at the excitation energy [48]. It is important to mention here that our observed red shift of the resonance frequency in the regime dominated by the lightning rod effect agrees well with the classical approach [49]. However, several recent quantum mechanical approaches calculated for sub-nanometer gap dimers predicted an opposite trend of the resonance frequency in the lightning rod effect regime [17,50,51,52].

To determine the spatial resolution, the full-width-at-half-maximum (FWHM) of the TERS EF profile in the middle of the gap is derived using a Gauss function. The simulated FWHM is then plotted as a function of the tip diameter in Figure 2c as indicated by black dots with the blue area representing the error bar obtained from the fitting. We purposefully set the error bar as 0.1 nm in the cases when the mathematical fit error goes below 0.1 nm, as the physical limit cannot go below this value. Initially, we can see an exponential increase of the FWHM as a function of tip diameter up to a value of 28 nm and then encounter a sharp drop. Interestingly, this sharp drop occurs at 28 nm where the maximum TERS EF has its minimum (see Figure 2b). This behavior indicates that the spatial confinement induced by the lightning rod effect is different from the FWHM of the plasmonic field stemming from the dipole-dipole interaction of LSPR mechanism. Comparing Figure 2b,c, it is thus evident that below a tip diameter of 28 nm, the lightening rod effect dominates the local field confinement mechanism, while above 28 nm of tip diameter the lightning rod effect becomes less significant due to the loss of the sharpness and the LSPR controls the plasmonic enhancement. The FWHM derived for a dipole-dipole system using Equation (3) is also plotted in Figure 2c shown by the red asterisks. As can be seen, our simulated FWHMs agree well with the estimated FWHMs beyond the tip diameter of 28 nm up to 110 nm. Further increasing the tip diameter size shows a smaller FWHM value compared with the estimation using Equation (3). This could be due to the decreasing electromagnetic energy loss of the structure in this range (see supplementary information Figure S1).

#### 3.1.2. Results on Tip Nanodisc Gap-Mode TERS Configuration Shown in Figure 1b

**Figure 3:**
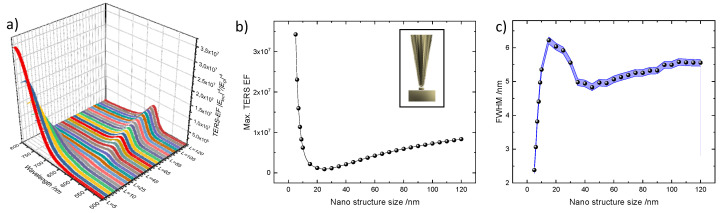
Simulation results when the tip is placed at the center of a nanodisc. (**a**) Spectral dependence of TERS EF, (**b**) maximum TERS EF, and (**c**) FWHM of the TERS EF profile. The blue shaded area presents the error bar of the FWHM. The tip-substrate geometry is shown in the inset of Figure 3b.

In this configuration, the gold tip diameter is kept constant at 80 nm. The diameter of gold nanodisc varies from 5 nm to 120 nm with a constant height of 20 nm. The simulated results for the model shown in Figure 1b are presented in Figure 3.

Figure 3a,b depict the spectral response of the TERS EF and maximum TERS EF as a function of nanodisc diameter with the tip at the center of the nanodisc. Similar to the previous configuration, the maximum TERS EF decreases rapidly as the nanodisc diameter increases from 5 nm to 25 nm due to the weakening of the coupling between the tip and the edges of the nanodisc as the edges move further away from the central tip position. However, further increasing the nanodisc diameter above 25 nm only leads to a moderate increase of the maximum TERS EF as can be seen in Figure 3b. This is because of the fixed tip diameter (80 nm), which dominates the resultant scattering cross-section of the image dipole formed at tip-substrate system. Hence, the overall TERS EF in the LSPR regime is weaker for the nanostructured substrate. However, in the lightning rod effect regime, the nanodisc significantly overpowers the gap-mode TERS EF (note that minimum tip diameter and nanodisc diameter in Figure 2b and Figure 3b are 2 nm and 5 nm, respectively). For example, the combination of a tip of 80 nm diameter and nanodisc diameter of 5 nm provides a TERS EF of 3.5 × 10^7^, which requires a 2 nm tip in the gap-mode TERS configuration. More importantly, to achieve this value, one needs to overcome some significant technological challenges related to the fabrication of such extremely sharp tips. On the contrary, thanks to the practical development, well-defined nanostructured substrates can be fabricated using electron beam lithography. Consequently, gold nanodiscs on gold films are favorable for achieving improved TERS EF.

To understand how the nanodisc diameter influences the spatial resolution, we extracted the FWHM of the spatial distribution of maximum TERS EF using the same procedure as above. The resultant FWHM vs. nanodisc diameter is plotted in Figure 3c. As can be seen, the FWHM for the nanostructured substrate configuration behaves differently compared to the gap-mode TERS configuration. Again, one can easily identify two regimes, which are dominated by the lightning rod effect and LSPR, respectively. The FWHM in the lightning rod effect regime is found to be between 2.3 nm and 6 nm within the range of the nanodisc diameter from 5 nm to 20 nm. Comparing Figure 2c and Figure 3c, we can see that the FWHM in this region in Figure 3c is larger than that in Figure 2c. This is because when the nanodisc diameter gets considerably smaller (in our simulation below 20 nm) than the tip diameter, the coupling between the tip and the two edges becomes stronger and thus enlarges the overall spatial distribution. In the region of nanodisc diameter from 20 nm to 40 nm, instead of a sharp drop shown in Figure 2c, we observe a gradual switching from the lightning rod effect regime to the LSPR regime since the increasing nanodisc diameter weakens the coupling between the tip and the edges of the nanostructures. Nevertheless, we can see the nanostructured substrate performs better in terms of TERS sensitivity if we compare the two configurations. For example, 6 nm of tip diameter in the first configuration provides a TERS EF of 1 × 10^7^ and a FWHM of 2 nm, whereas a nanodisc diameter of 5 nm with a tip diameter of 80 nm gives a TERS EF of 3.5 × 10^7^ and a FWHM of 2.3 nm.

**Figure 4:**
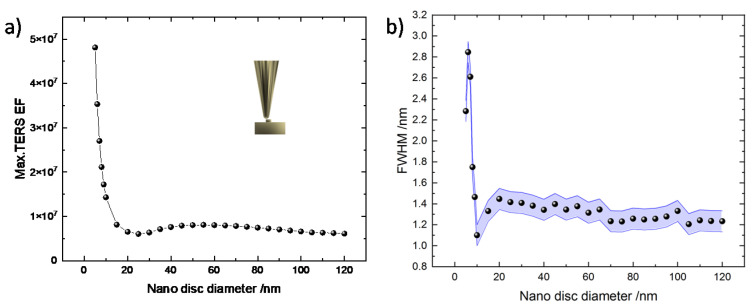
Simulation of TERS EF when the tip is placed at the edge of a nanodisc. (**a**) Maximum TERS EF and (**b**) FWHM of the TERS intensity. The geometry is presented in the inset of Figure 4a. The blue shaded area presents the error bar of the FWHM in Figure 4b.

In our previous work, we demonstrated experimentally that edges of nanostructures can produce even stronger TERS sensitivity [3,53,54,55]. Therefore, the nanostructured substrate configuration was further tested to understand how the dimension of the nanostructure influences the TERS sensitivity when the tip is located at the edges. The calculated max. TERS EF and the FWHM as a function of the nanodisc diameter are presented in Figure 4a,b. The spectral dependence of the TERS EF can be found in the supplementary information (Figure S2). It is important to mention here that we kept the curvature of the edges at 2 nm for all nanodiscs considered in the simulations.

Comparison between Figure 3b and Figure 4a reveals that the maximum TERS EF is further amplified especially in the range of nanodisc diameters from 5 to 10 nm where the lightning rod effect is assumed to be the strongest. The impact of the lightning rod effect is observable up to 20 nm of nanodisc diameter, while further increasing nanodisc diameter does not influence the TERS EF significantly. This is also visible in Figure 4b, where the FWHM remains constant within the fitting uncertainty above 20 nm of nanodisc diameter. However, when decreasing the nanodisc diameter below 10 nm, we observe an increase of the FWHM, most likely due to coupling with the opposite edge of the nanodisc. The smallest FWHM achieved in this configuration is 1.1 nm for a nanodisc diameter of 10 nm. This is certainly a further improvement compared to the two other configurations discussed above. More importantly, the TERS EF of 1.6 × 10^7^ achieved at this nanodisc diameter for a tip placed at the edge is significantly better than the two configurations discussed above with equivalent dimensions. For 6 nm of tip diameter and 5 nm of nanodisc diameter, we obtain a TERS EF and FWHM of 1 × 10^7^ and 2 nm (tip on flat gold thin film), 3.5 × 10^7^ and 2.3 nm (tip at the centre of nanodisc), and 5 × 10^7^ and 1.8 nm (tip at the edge of nanodisc), respectively. Therefore, our 2D simulations clearly demonstrate a path to improve the TERS sensitivity, which is critical and highly sought after in optical nanospectroscopy.

### 3.2. Experimental Results and 3D Simulation

**Figure 5:**
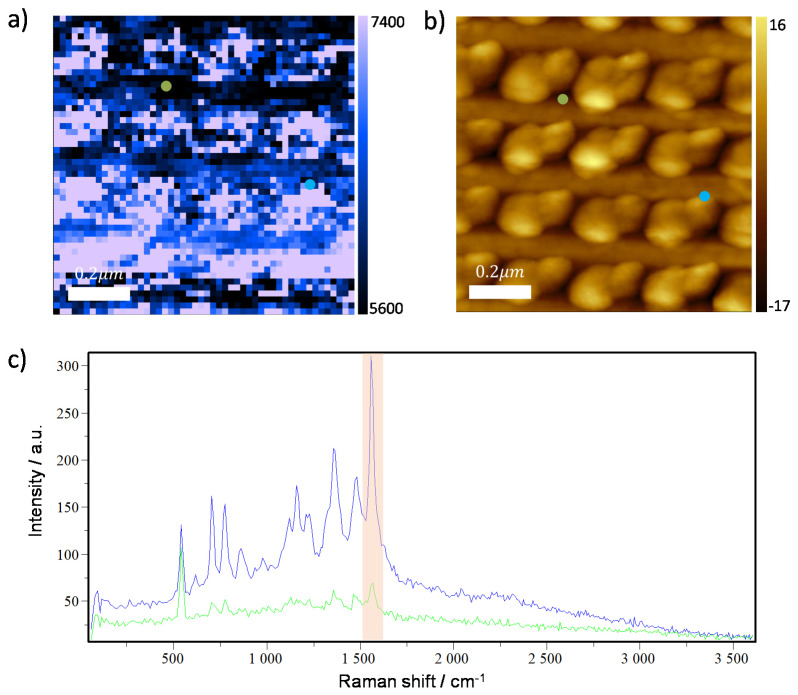
TERS experiment on the reference nanodisc—modified gold substrate. (**a**) TERS map, (**b**) Atomic-force microscopy AFM topography, and (**c**) corresponding Raman spectra taken from two different positions marked by blue and green dots in the TERS map. The TERS map is created using the most intense peak around 1535 cm^−1^ as shown by the red shaded region in Figure 5c.

**Figure 6:**
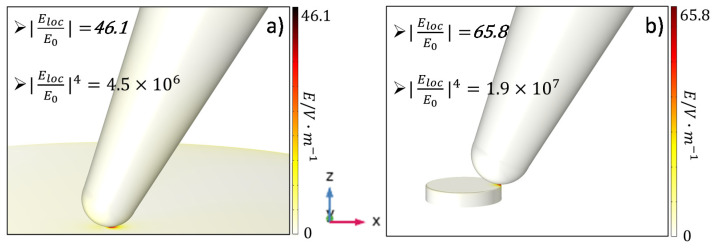
3D simulation results of (**a**) Au tip with diameter of 80 nm on the flat gold substrate; (**b**) Au tip with diameter of 80 nm at the edge of a 20 nm high Au nanodisc with disc diameter of 100 nm. The electric field EF and corresponding TERS EF are presented in the figures respectively.

TERS experiments are performed on a nanostructured substrate consisting of a gold nanodisc array on a gold coated silicon substrate to compare with our simulation. An ultrathin (~2 nm) film of cobalt phthalocyanine (CoPc) is deposited on this plasmonic substrate as a Raman probe. Figure 5a,b present the TERS map and AFM topography taken simultaneously using a home-made gold coated TERS tip. The step size of the measurements is 10 nm. Two representative TERS spectra, one on the flat gold surface (green dots in Figure 5a,b) and another one at the edge of the gold nanodisc (blue dots in Figure 5a,b) are shown in Figure 5c with corresponding colors. The TERS map is created for the most intense CoPc Raman peak at 1535 cm^−1^ as shown by the red shaded area in Figure 5c. As can be seen in Figure 5a, the highest TERS enhancement originates predominantly from the edges of the nanodiscs as predicted by the simulation above. The modified AFM topography in Figure 5b is most likely due to the imperfect profile of the tip apex with multiple clusters contributing to the topography convolution. Nevertheless, from Figure 5a it is evident that the imperfect profile of the apex has less influence on the TERS map, meaning that coupling between the gold clusters and the nanodisc dictates the TERS mapping. Comparing the TERS spectra shown in Figure 5c, it is evident that peak intensities of CoPc from the nanodisc edge are stronger than from the flat gold. For example, the peak intensity of the phonon mode centered at 1535 cm^−1^ is about 4.1 times larger than that from the flat gold surface. Our 3D simulations with respect to these two geometries with the same excitation of 638 nm are shown in Figure 6. It can be seen that the electric field enhancement factor in Figure 6a, representing the geometry of the tip on flat gold, is 46.1, corresponding to the TERS EF of $4.5\times 10^{6}$. On the other hand, the electric field EF shown in Figure 6b representing the geometry of the tip on the edge of nanodisc, is 65.8, corresponding to the TERS EF of $1.9\times 10^{7}$. Thus, the relative EF between the flat gold and the nanodisc edge is calculated to be 4.2. This is in a very good quantitative agreement to the TERS experimental result.

In addition, TERS mapping is also performed on other gold nanostructured substrates consisting of monolayer MoS_2_ on gold nanodisc arrays on Si (see Figure S3). In this experiment, MoS_2_ is used as the Raman probe. Here, it is even more evident that the maximum TERS enhancement originates from the edge of the nanodiscs, demonstrating good agreement with the current simulation results. Furthermore, our previous study also confirmed qualitatively the result from the 2D simulation [3,55]. However, we performed a detailed systematic computational study in the present investigation and report the influence of the coupling behaviour between the tip and the plasmonic substrate on TERS sensitivity, which is missing in the previous work [3,55].

A comparison on the TERS EF between the 2D and 3D simulations for two specific experimental geometries shown in Figure 6 is demonstrated in Table 1. The tip diameter of 80 nm and the nanodisc diameter of 100 nm are set for both 2D and 3D simulation. The excitation wavelength is set as a *p*-polarized 638 nm with light polarized along the tip long axis. Nevertheless, there are some different settings between the 2D and 3D configurations; for instance, the tip is placed perpendicular to the surface of substrate in 2D geometry, while it is 25° tilted from the surface normal in 3D.

Note that the plasmonic resonance depends on the geometry of the system. Therefore, it is not necessarily true that both 2D and 3D plasmonic response will be in resonance at the same excitation wavelength (638 nm in the present investigation). As can be seen from Table 1, both 2D and 3D simulations shows the same qualitative trends that the TERS EF is higher when the tip is placed on the edge of the nanodisc compared with when the tip is placed on flat gold. However, the relative scale is around 1.1 for 2D and 4.1 for 3D simulations. Even though we see a perfect quantitative agreement between the 3D simulation results and experiments, the 2D simulation does offer a qualitative agreement with experiment as well. Considering the huge computational cost of any 3D simulation for large data set, 2D simulation is a very powerful tool providing a fast and systematic understanding for any plasmonic systems.

## 4. Summary

In summary, we investigate the possibility of improving TERS sensitivity via a systematical 2D FEM simulation study for two configurations. The first one consists of a gold conical tip with various diameters placed on a flat gold thin film representing the conventional gap-mode TERS and the other one consists of a gold conical tip with a fixed diameter of 80 nm placed on a gold nanodisc modified gold thin film. For the second configuration we investigated two possibilities—one with the tip placed at the center of the nanodisc and the second with the tip placed at the edge of the nanodisc. Our simulations clearly demonstrate that the nanodisc substrate provides better TERS sensitivity (both TERS EF and spatial resolution) compared to the conventional gap-mode TERS. Additionally, we also observed that the best TERS performance (strong improvement of both TERS EF and spatial resolution) can be achieved when the tip is placed at the edge of the nanostructures. We also conducted TERS experiments and the corresponding 3D simulation to validate our simulation results and found very good agreement between them. Our model can also be adopted for any other materials. Our results will help to understand and design the high resolution TERS experiments, which is highly pursued in optical nanospectroscopy.

## Figures and Tables

**Table 1:** Comparison on TERS EF between 2D and 3D simulation results for the two specific geometries representing the two experimental configurations.

	Tip on Flat Gold	Tip on Edge of Nanodisc
2D simulation	$7.7\times 10^{6}$	$8.2\times 10^{6}$
3D simulation	$4.5\times 10^{6}$	$1.9\times 10^{7}$

## Data Availability

The data presented in this study are available on request from the corresponding author.

## Supplementary Materials

The following are available online at https://www.mdpi.com/2079-4991/11/2/376/s1, Figure S1: Max. TERS EF and EM loss energy density vs. tip size, Figure S2: Spectral dependence of TERS EF when the tip placed at the edge of the nanodisc, and Figure S3: TERS on monolyer MoS_2_ on gold nanodisc arrays.
