# Supplementary material for: Understanding the Role of Different Substrate Geometries for Achieving Optimum Tip-Enhanced Raman Scattering Sensitivity
Source: Nanomaterials (Basel). 2021 Feb 2;11(2):376. doi: 10.3390/nano11020376 (PMC7913005; doi:10.3390/nano11020376)
Supplement: Supplementary file 1 [file nanomaterials-11-00376-s001.pdf]

nanomaterials

MDPI

Article

Understanding the Role of Different Substrate Geometries for Achieving Optimum Tip-Enhanced Raman Scattering Sensitivity

Lu He, Mahfujur Rahaman *, Teresa I. Madeira, and Dietrich R.T Zahn

Semiconductor Physics, Chemnitz University of Technology, D-09107 Chemnitz, Germany; lu.he@s2017.tu-chemnitz.de (L.H.); Teresa.madeira@physik.tu-chemnitz.de (T.I.M.); zahn@physik.tu-chemnitz.de (D.R.T.Z.)
* Correspondence: mahfujur.rahaman@physik.tu-chemnitz.de

Figure S1: Max. TERS EF and EM loss energy density *vs* tip size

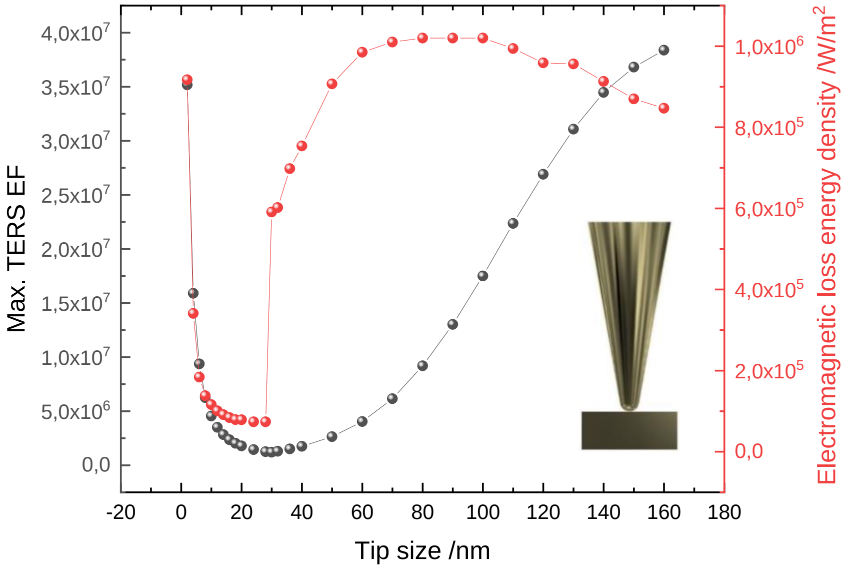

Figure S1. Max. TERS EF and electromagnetic loss energy density for the configuration shown in the inset and Figure 2 in the main text.

Figure S2: Spectral dependence of TERS EF when the tip placed at the edge of the nanodisc

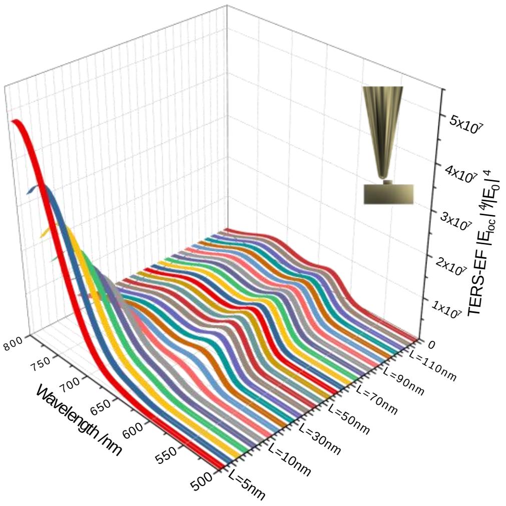

Figure S2. Spectral dependence of the TERS EF for the configuration shown in the inset. The tip diameter is fixed at 80 nm and the nanodisc size was varied in the simulation. The max. TERS EF and the FWHM as a function of nanodisc diameter is presented in Figure 4 in the main text.

Figure S3: TERS on monolayer MoS_2 on gold nanodisc arrays

TERS measurements of monolayer MoS_2 deposited on gold nanodisc arrays are presented in Figure S3. Gold naodiscs were fabricated on Si substrate using electron beam lithography and the monolayer MoS_2 was transferred onto the plasmonic substrate using deterministic dry transfer method. Details of the sample preparation can be found in ref. [1]. The dimension of the gold discs are: diameter of 150 nm and height of 50 nm. A home-made gold tip was used to acquire the TERS signal under the excitation of 785 nm. The laser power was 100 μW and the acquisition time was 0.3 s. TERS map forms ring like shape resembling the periphery of the nanodiscs, in agreement to the 2D and 3D simulations presented in the main text.

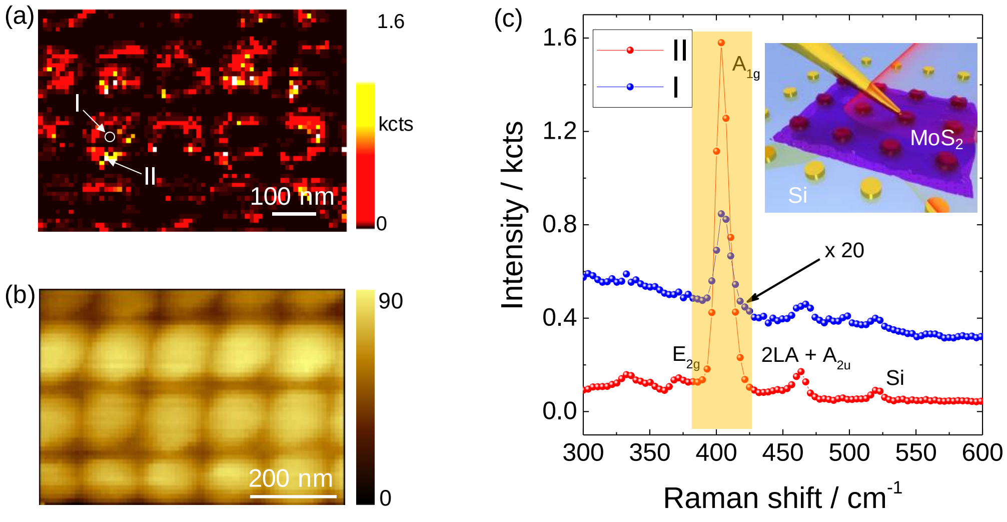

Figure S3. TERS measurement on gold nanodisc arrays on Si. Monolayer MoS2 was used as the probing material. (**a**) The TERS map, (**b**) corresponding AFM topography taken simultaneously, and (**c**) two representative TERS spectra, one at the edge of the gold disc and other at the center of the disc as shown in the TERS map. The schematic of the TERS measurement is shown in the inset of (**c**).

Reference:

1. Milekhin A G, Rahaman M, Rodyakina E E, Latyshev A V, Dzhagan V M, Zahn D R T. Giant gap-plasmon tip-enhanced Raman scattering of MoS_2 monolayers on Au nanoclusters arrays. Nanoscale **2018**, *10*, 2755.
